# Amino acids-incorporated nanoflowers with an intrinsic peroxidase-like activity

**DOI:** 10.1038/srep22412

**Published:** 2016-03-01

**Authors:** Zhuo-Fu Wu, Zhi Wang, Ye Zhang, Ya-Li Ma, Cheng-Yan He, Heng Li, Lei Chen, Qi-Sheng Huo, Lei Wang, Zheng-Qiang Li

**Affiliations:** 1 Key Laboratory of Molecular Enzymology and Engineering of Ministry of Education, College of Life Science, Jilin University, Changchun, 130012, China; 2 College of Life Science, Jilin Agricultural University, Changchun, 130118, China; 3 State Key Laboratory of Inorganic Synthesis and Preparative Chemistry, College of Chemistry, Jilin University, Changchun, 130012, China; 4 The Third Hospital of Jilin University, Changchun, 130033, China

## Abstract

Functional molecules synthesized by self-assembly between inorganic salts and amino acids have attracted much attention in recent years. A simple method is reported here for fabricating hybrid organic–inorganic nanoflowers using copper (II) ions as the inorganic component and natural amino acids as the organic component. The results indicate that the interactions between amino acid and copper ions cause the growth of the nanoflowers composed by C, N, Cu, P and O elements. The Cu ions and Cu(AA)_n_ complexes containing Cu-O bond are present in the nanoflowers. The nanoflowers have flower-like porous structure dominated by the R groups of amino acids with high surface-to-volume ratios, which is beneficial for exerting its peroxidase-like activity depending on Fenton-like reaction mechanism with ABTS and Rhodamine B as the substrates. It is expected that the nanoflowers hold great promise as enzyme mimics for application in the field of biosensor, bioanalysis and biocatalysis.

Numerous works have been devoted to the synthesis and characterization of the nano-structured materials^1^,^2^,^3^,^4^,^5^,^6^. These studies are paving the way from the nano- to macro-scopic world. Among of the nano-structured materials, the bio-inspired materials with micro- and nano-scale have been proposed as a big breakthrough on the design of advanced functional materials and have attracted much attention in recent years due to the huge advantage of the bio-molecules in directing and assembling the superstructures^7^. For example, Zare and co-workers have successfully prepared protein-inorganic hybrid nanostructures with flower-like shapes in 2012^8^. When an enzyme is used as the protein component, the hybrid nanoflower exhibits enhanced enzymatic activity and stability compared with the free enzyme. This is attributed to the high surface area and confinement of the enzymes in the nanoflowers. The peptide nanoparticles have also been prepared *via* different routes and exhibit excellent function as catalyst or as a basis of smart and responsive materials that can sense or control diverse biological events^9^. However, it still remains a big challenge for scientists to assemble bioinorganic hybrid structures into the complex hierarchical architectures for their wide potential applications^10^.

Amino acids are considered as the major building blocks of all naturally occurring peptides and proteins. Furthermore, their side chains vary a lot from each other, making them have the potential usefulness in chiral molecular recognition and selection processes^11^,^12^. Here, we describe a simple method for fabricating the hybrid organic–inorganic nanoflowers using copper (II) ions as the inorganic component and natural amino acids as the organic component under the mild conditions.

## Results and Discussion

**Figure 1:**
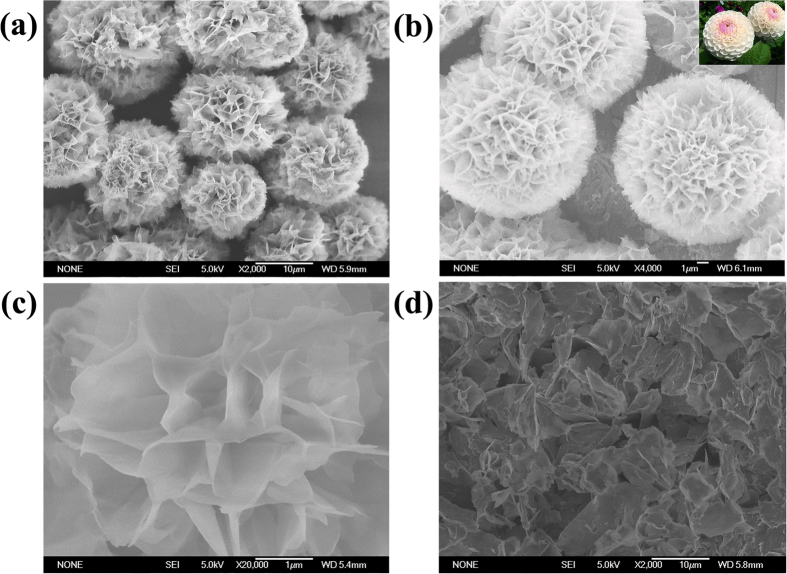
SEM of the hybrid organic–inorganic nanoflower prepared from Asn. (**a**) SEM image of the nanoflowers; (**b**) a single nanoflower; (**c**) High-resolution SEM image of the porous structure of the petals; (**d**) the disordered fragments formed without Asn; the inset of (**b**) the Dahlia in nature.

In a typical experiment, the hybrid organic-inorganic nanoflowers were prepared by mixing 20 μL of aqueous CuSO_4_ solution (120 mM) and 3 mL of phosphate buffer (pH 7.4) containing asparagine (Asn) (60 μg) at 25 °C. After 24 hours, a blue precipitate with porous, flower-like structures was fabricated. Figure 1 presents the scanning electron microscope (SEM) images of the nanoflower prepared from Asn. Figure 1a,b reveal that the samples consist of large quantities of flower-like nanostructures with diameters in the range of 10–15 μm. The morphology of the nanoflower closely resembles the shape of Dahlia in nature (the insets of Fig. 1b). As shown in Fig. 1c, the nanoflowers have hierarchical structures with high surface-to-volume ratios. Figure 1d demonstrates that disordered fragments are formed without Asn. These results certify that amino acids play a crucial role in the formation of nanoflowers. The SEM images prepared from other 19 kinds of natural amino acids were listed in the supporting materials (Fig. S1). The morphology of the nanoflowers prepared from different natural amino acids vary slightly due to their various side chains, which may affect the coordination geometry of the copper (II) complexes^13^.

**Figure 2:**
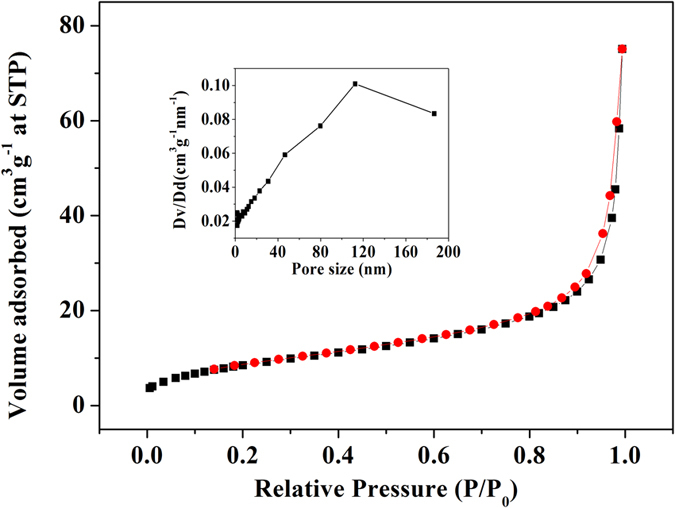
Nitrogen adsorption-desorption isotherm and the pore size distribution curve (inset) for the nanoflowers.

It is known that Brunauer-Emmett-Teller (BET) method can show the exact value of the surface area of samples. Therefore the BET surface area of Asn-incorporated nanoflowers was determined by a nitrogen adsorption-desorption measurement. The nitrogen isotherm of the Asn-incorporated nanoflowers is a type IV isotherm with a narrow hysteresis loop in the range of 0.8–1.0 *P/P*_*0*_ (Fig. 2). Surface area measurements reveal that Asn-incorporated nanoflowers has a BET surface area of 32.0 m^2^ g^−1^ and an average pore size of 112.5 nm (the inset of Fig. 2).

**Figure 3:**
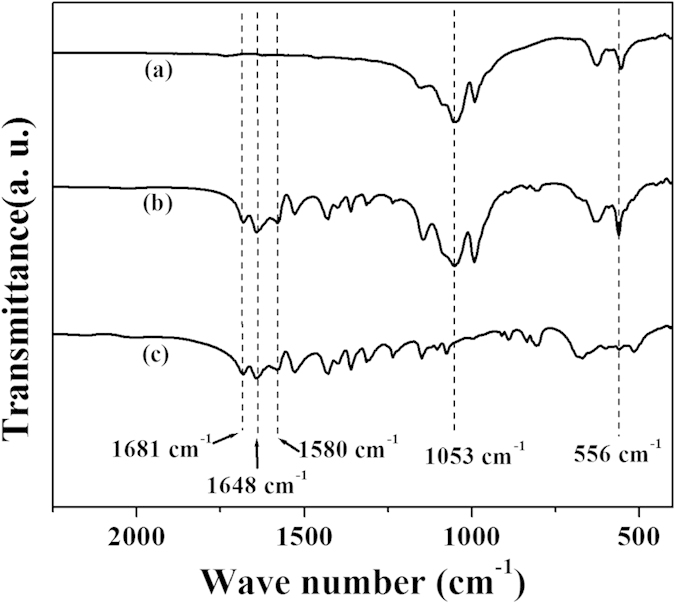
FTIR of the hybrid organic–inorganic nanoflower prepared from Asn. (**a**) Cu_3_(PO_4_)_2_ matrices; (**b**) Asn-incorporated nanoflower; (**c**) Asn.

The FTIR spectrums of the Cu_3_(PO_4_)_2_ matrices, Asn and Asn-incorporated nanoflower were shown in Fig. 3. The peaks at 1053 cm^−1^ and 556 cm^−1^ are assigned to the vibrations of PO_4_^3−^ (curve a-b in Fig. 3)^14^. The peaks at 1681 cm^−1^, 1648 cm^−1^ and 1580 cm^−1^ are ascribed to the vibrational spectra of the Asn (curve b-c in Fig. 3)^15^. These results verify the presence of Asn in the nanoflower.

**Figure 4:**
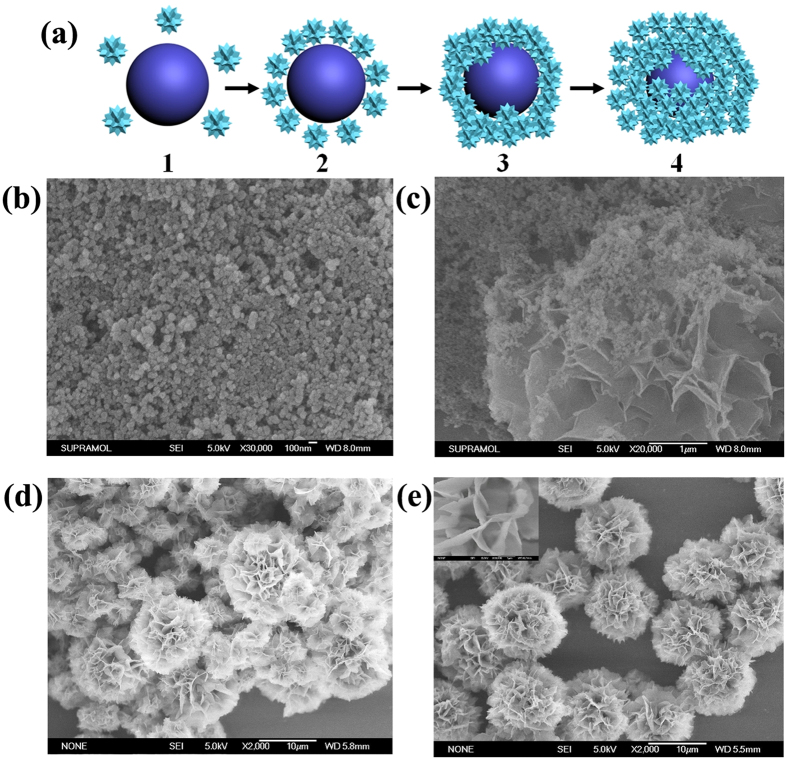
The possible mechanism (**a**) and the SEM images of nanoflower at different growth stages. (**b**) 0h; (**c**) 0.5h; (**d**) 2h; (**e**) 24h.

**Figure 5:**
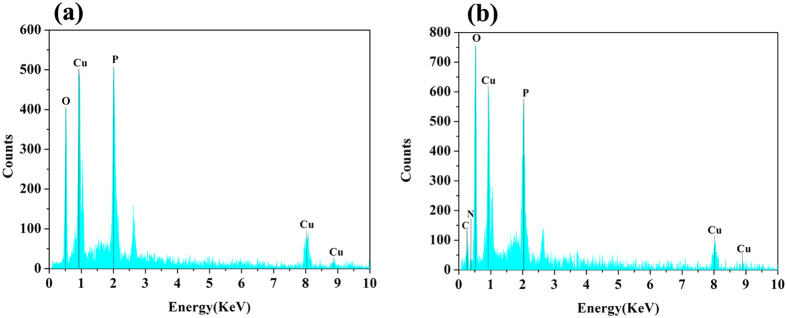
EDS pattern of Asn-incorporated nanoflower at different growth stages. (**a**) 0h; (**b**) 24h.

SEM was used to monitor the different growth stages of the nanoflower and a possible mechanism is proposed in Fig. 4a. At the first stage (step 1), the Cu(AA)_n_ complexes (AA, amino acid), which can be favorable for intermolecular interactions^16^, are formed in solution predominantly through the coordination facility of amide groups and carboxyl groups. Meanwhile, some primary crystals of copper phosphate are also formed and suspended in the solution or precipitated at the bottom (Fig. 4b). The composition of the precipitate at the first stage was confirmed by its energy dispersive x-ray spectroscopy (EDS) spectrum (Cu/P = 3:2), which verifies the existence of copper phosphate (Fig. 5a). The formed crystals of copper phosphate may provide the original core of the nanoflower. In the second growth step, the Cu(AA)_n_ complexes attack the copper phosphate core by ion exchange and form a novel coordination core (step 2, Fig. 4c). In the third stage, (step 3) the Cu(AA)_n_ complexes in solution continue to attack the newly formed core repeatedly through the intermolecular interactions between Cu(AA)_n_ and the core. This process can enlarge the agglomerates. Some small flowers in the process of growing up can be observed in this growth stage (Fig. 4d). In the last stage (step 4, Fig. 4e), the mature nanoflower in blossom can be obtained. The ligand-ligand interactions in Cu(AA)_n_ complexes and the steric effect of the side chain may cause the separate petals to appear and the anisotropic growth results in complete formation of a branched flower-like structure (the inset of Fig. 4e). The EDS analysis of the mature nanoflower indicates the samples consist mainly of C, N, Cu, P and oxygen (Fig. 5b). The atom ratio of Cu:P is approximately 3:2. The EDS spectrum of the nanoflower reveals that the nanoflowers are composed of Cu_3_(PO_4_)_2_ crystals dispersed into the organic amino acid component.

**Figure 6:**
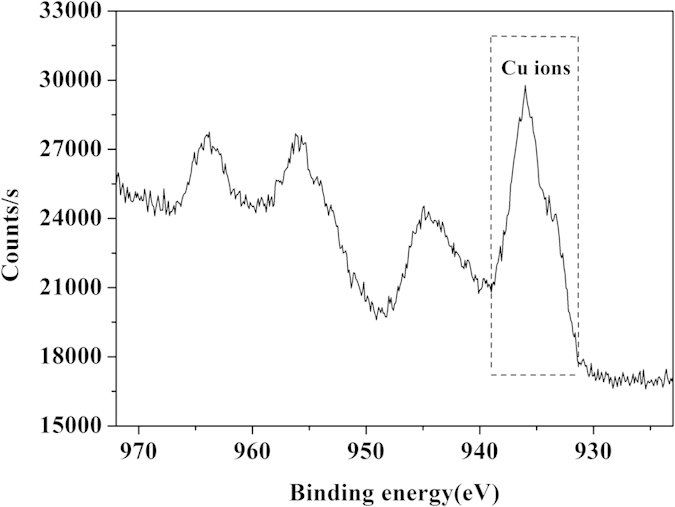
XPS spectrum of Asn-incorporated nanoflower.

**Figure 7:**
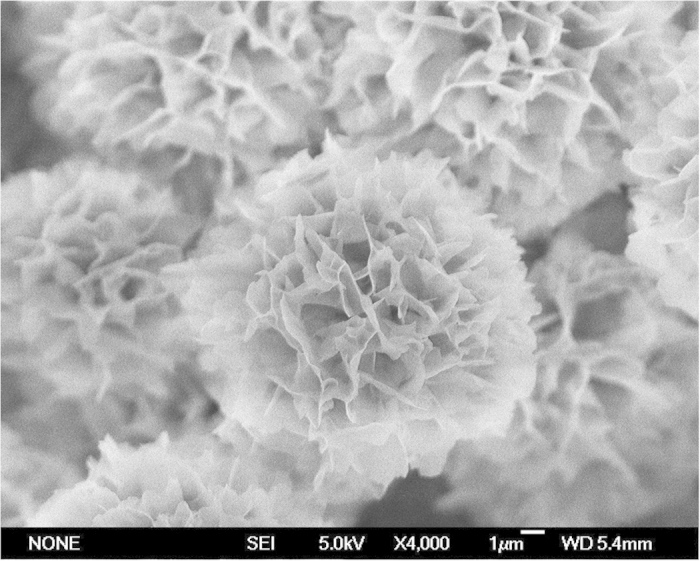
SEM of the hybrid organic–inorganic nanoflower prepared from Asn through another route.

In order to verify this proposed mechanism, Raman spectroscopy was used to identify the presence of Cu(AA)n complexes in the nanoflowers at the level of molecular bonds. The Raman spectra of the nanoflowers show three peaks at 297 cm^−1^ (Ag), 352 cm^−1^ (Bg^1^) and 642 cm^−1^ (Bg^2^) which are assigned as characteristic peaks of Cu-O bond (Figure S2)^17^,^18^,^19^. As for Cu_3_(PO_4_)_2_, CuSO_4_ (Curve u and v of Figure S2) and 20 kinds of amino acids (Figure S3), no characteristic peaks for Cu-O bond can be observed. It’s known that copper-amino acid complexes contain Cu-O bond formed by the coordination between Cu atom and the carboxyl O atom from amino acid^20^,^21^,^22^. Hence, these results indicate that the peaks at 297 cm^−1^ (Ag), 352 cm^−1^ (Bg^1^) and 642 cm^−1^ (Bg^2^) in Raman spectra of the nanoflowers is due to the contribution of Cu-O bond from Cu(AA)n complexes in the nanoflowers. X-ray photoelectron spectroscopy (XPS) spectrum of Asn-incorporated nanoflowers also demonstrates that the existence of the Cu ions in the nanoflower (Fig. 6). Moreover, we have designed another assay to fabricate the nanoflowers. The crystals of copper phosphate were prepared firstly by mixing 20 μL of aqueous CuSO_4_ solution (120 mM) and 3 mL of phosphate buffer (pH 7.4, 0.05M) at 25 °C. The precipitate was collected and added into 3 mL of aqueous solution containing 60 μg Asn and 20 μL of aqueous CuSO_4_ solution (120 mM). After 24 h, the nanoflower appeared and the image was shown in Fig. 7. This assay suggests that Cu(AA)_n_ in the solution can attack the phosphate copper and fabricate the nanoflower.

It’s known that copper compound possess intrinsic peroxidase-like activity in the presence of H_2_O_2_^23^. Therefore, the peroxidase-like activities of the Cu(AA)n complexes in amino acid-incorporated nanoflowers were investigated. As can be seen from Figure S4a, Asn-incorporated nanoflowers catalyze one-electron oxidation of ABTS into the radical cation ABTS^•+^ which has a strong absorption at 417 nm in the presence of H_2_O_2_. Asn-incorporated nanoflowers alone or H_2_O_2_ alone can not yield ABTS^•+^. The peroxidase-like activity of Asn-incorporated nanoflowers significantly increases with the increase of H_2_O_2_ and the nanoflowers concentration in a certain range (Figure S4b and S4c), which is in agreement with the performance of protein-incorporated nanoflowers^24^. Based on the reaction mechanism of Fenton-like reaction^25^,^26^, Qu *et al.* speculated the protein-incorporated nanoflowers exhibited peroxidase-like activity depending on the reaction mechanism of Fenton-like reaction^24^. They proposed that Cu^2+^ ions reacted with hydrogen peroxide to form Cu^1+^ ions (Formula 1), and then the highly-reactive hydroxyl radical formed from the reaction between Cu^1+^ ions in the nanoflowers and hydrogen peroxide (Formula 2). The free hydroxyl radical produced from the copper-redox cycle triggered the oxidation of ABTS. In this work, the catalytic mechanism of amino acid-incorporated nanoflowers is possible similar to that of protein-incorporated nanoflowers^24^.


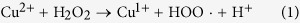



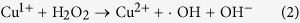


As can be seen in Figure S5 , the peroxidase-like activities of 20 kinds of amino acid-incorporated nanoflowers are higher than that of Cu_3_(PO_4_)_2_ crystal. When the same amount of Cu^2+^ ions was employed to oxidize ABTS, no peroxidase-like activity could be detected (Figure S6). The peroxidase-like activities of amino acid-incorporated nanolfowers are listed on Table S1. Depending on the difference of R groups, the peroxidase-like activities of amino acid-incorporated nanoflowers are compared and the results are as follows: “Positively charged R groups >Nonploar, aliphatic R groups >Aromatic R group >polar, uncharged R groups >negatively charged groups” (Table S1). The results indicate that the peroxidase-like activities of amino acid-incorporated nanofowers are affected by the electronegativity of R groups of amino acids. The results suggest that the electron-donating group in positively charged R groups is helpful for inducing the appearance of Cu^1+^ ions in the nanoflowers.

It’s can be seen from Figure S7a and Figure S7b that Asn-incorporated nanoflowers successfully decompose Rhodamine B in the presence of H_2_O_2_ after 6 hours incubation. Either the nanoflower alone or H_2_O_2_ alone can not remove Rhodamine B (Figure S7a and Figure S7b). The decomposition efficiencies increase with the increase of the concentration of nanoflowers or H_2_O_2_ within a certain range (Figure S7c and Figure S7d). Other 19 amino acid-incorporated nanoflowers also can decompose Rhodamine B and its specific activities are higher than that of Cu_3_(PO_4_)_2_ crystal (Table S2). After the same amount of Cu^2+^ ions was employed to incubate Rhodamine B for 6 hours, the absorption of Rhodamine B at OD_550_ did not drop (Figure S8). On the basis of the difference of R groups, the specific activities of amino acid-incorporated nanoflowers using Rhodamine B as the substrate are compared and the results are as follows: “Positively charged R groups > Nonploar, aliphatic R groups > Aromatic R group > polar, uncharged R groups > negatively charged groups” (Table S2).

**Figure 8:**
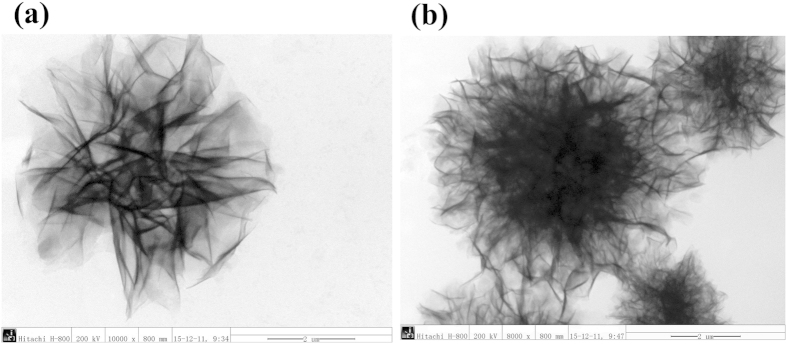
TEM of the hybrid organic–inorganic nanoflower prepared from Asn (**a**) or Lys (**b**).

In order to investigate the effect of R groups on the structure of the nanoflowers, the transmission electron microscopy (TEM) images of Asn-incorporated nanoflowers and Lys-incorporated nanoflowers were compared. As compared with Asn-incorporated nanoflowers, Lys-incorporated nanoflowers exhibit more compact structures (Fig. 8), which may be due to the effect of the polarity of R group.

In summary, we have developed a simple method to fabricate nanoflowers using copper (II) ions as the inorganic component and natural amino acids as the organic component under mild conditions. The functional characterization indicates that amino acid-incorporated nanoflowers exert its catalytic activity on the basis of the catalytic principle of Fenton’s like reagents. Owing to the existence of Cu ions, the amino acids-incorporated nanoflowers exhibit intrinsic peroxidase-like activity by the self-assembly between amino acids and copper phosphate, while the protein-incorporated nanoflowers only present the enzymatic activity of protein component. We expect that these amino acid-inorganic hybrid nanoflowers will have important applications in biosensors, bioanalytical devices, pharmaceutical applications and industrial biocatalysis.

## Methods

### Synthesis of amino acid-incorporated nanoflowers

At first, 60 ml of amino acid solution (1 mg ml^−1^) was added to 3 l of PBS solution (50 mmol l^−1^, pH 7.4), followed by the addition of 20 ml of CuSO_4_ solution (120 mmol l^−1^). Then, the mixture was incubated at 25 °C for three days. The blue product could be found at the bottom of the flash. Finally, the blue product was collected by centrifugation (12,000 rpm for 20 min) and washed by deionized water for three times.

### Characterization of amino acid-incorporated nanoflowers

The morphologies of the samples were observed by a JSM-6700F electron microscope (JEOL, Japan) with an acceleration voltage of 30 kV. The FTIR spectrums of the samples were surveyed using Nicolet 5700 FTIR spectrometer with a resolution of 4 cm^−1^ through KBr method. Raman spectra were measured on InVia Raman Microscope. Excitation was by means of the 488 nm line of an argon ion laser with an output power in the range of 200 to 300 mw. The instrument is equipped with a microscope with a focal spot size in the range of a few micrometers. The adsorption-desorption isotherms of nitrogen were measured at 77 K by using a Micromeritics ASAP 2420 analyzer. The EDS spectra (JEOL JSM-6700F, Japan) were also used to analyze the composition of the samples. XPS spectrum was collected on a Thermo ESCALAB 250. The morphology and structure of the samples were analyzed by TEM using a FEI Tecnai G2 F20 s-twin D573 operated at 200 kV.

### Peroxidase-like activity measurements

The peroxidase-like activity of the amino acid-incorporated nanoflower (20 μg/ml) were investigated through the catalytic oxidation of the peroxidase substrate ABTS (1 mM) in the presence of 25 mM H_2_O_2_. All the reactions were incubated in phosphate buffer (pH 4.0, 10 mM) and monitored by the Shimadzu UV-2550 spectrometer at 417 nm. The specific activity (μmol/mg/min) was defined as the amount (in micromoles) of ABTS^•+^ produced per minute per milligram of the nanoflowers.

### Rhodamine B assay

The specific activity of the amino acid-incorporated nanoflower (20 μg/ml) were investigated using Rhodamine B (12.5 μg/mL) as a substrate in the presence of 100 mM H_2_O_2_. All samples were incubated with Rhodamine B in phosphate buffer (pH 4.0, 10 mM) at 37 °C and monitored by the Shimadzu UV-2550 spectrometer at 550 nm. The specific activity (μmol/mg/min) was defined as the amount (in micromoles) of Rhodamine B consumed per minute per milligram of the nanoflowers.

## Additional Information

**How to cite this article**: Wu, Z.-F. *et al.* Amino acids-incorporated nanoflowers with an intrinsic peroxidase-like activity. *Sci. Rep.* **6**, 22412; doi: 10.1038/srep22412 (2016).

## Supplementary Material

Supplementary Information
