# Supplementary material for: Amino acids-incorporated nanoflowers with an intrinsic peroxidase-like activity
Source: Sci Rep. 2016 Mar 1;6:22412. doi: 10.1038/srep22412 (PMC4772475; doi:10.1038/srep22412)
Supplement: Supplementary Information [file srep22412-s1.pdf]

Amino acids-incorporated nanoflowers with an intrinsic peroxidase-like activity

Zhuo-Fu Wu[1,2,+], Zhi Wang[1,+], Ye Zhang[3], Ya-Li Ma[3], Cheng-Yan He[4], Heng Li[1], Lei Chen[1], Qi-Sheng Huo[3], Lei Wang[1,*] & Zheng-Qiang Li[1,*]

[1]Key Laboratory of Molecular Enzymology and Engineering of Ministry of Education, College of Life Science, Jilin University, Changchun, 130012, China, [2]College of Life Science, Jilin Agricultural University, Changchun, 130118, China, [3]State Key Laboratory of Inorganic Synthesis and Preparative Chemistry, College of Chemistry, Jilin University, Changchun, 130012, China, [4]The Third Hospital of Jilin University, Changchun, 130033, China.

[*]Correspondence and requests for materials should be addressed to L.W. (w_lei@jlu.edu.cn) or Z.L. (lzq@jlu.edu.cn)

[+]These authors contributed equally to this work.

Experiment section

Reagent and materials: 2, 2-azinobis (3-ethylbenzothiozoline)-6-sulfonic acid (ABTS) and Rhodamine B (RhB) were obtained from Sigma-Aldrich. KBr (spectral grade) was obtained from BDH Co. (Poole, UK). All chemicals and reagents were of analytical grade. All aqueous solutions were prepared with Milli-Q water.

Electronic Supplementary Information

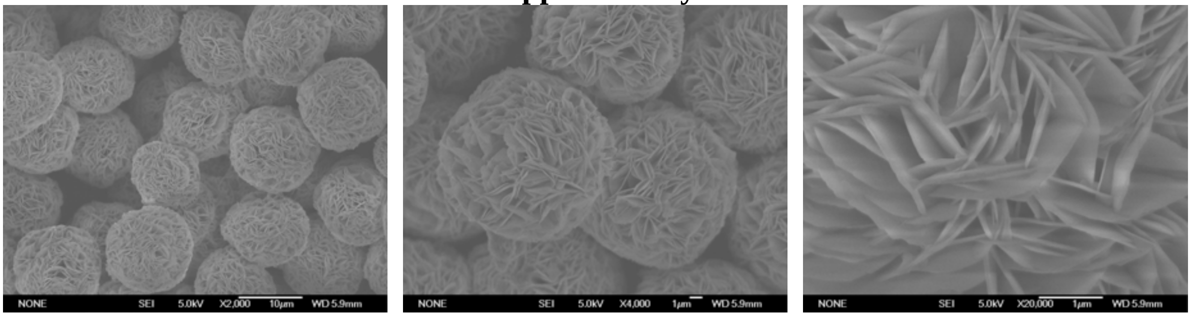

Cysteine-incorporated nanoflower

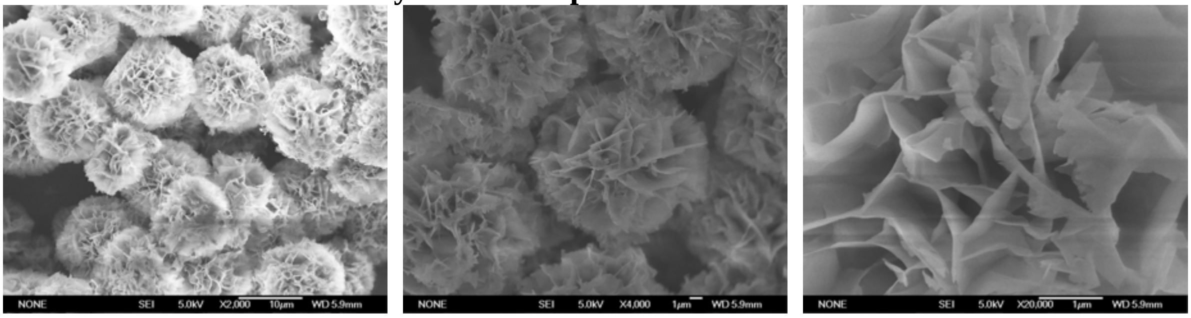

Glycine-incorporated nanoflower

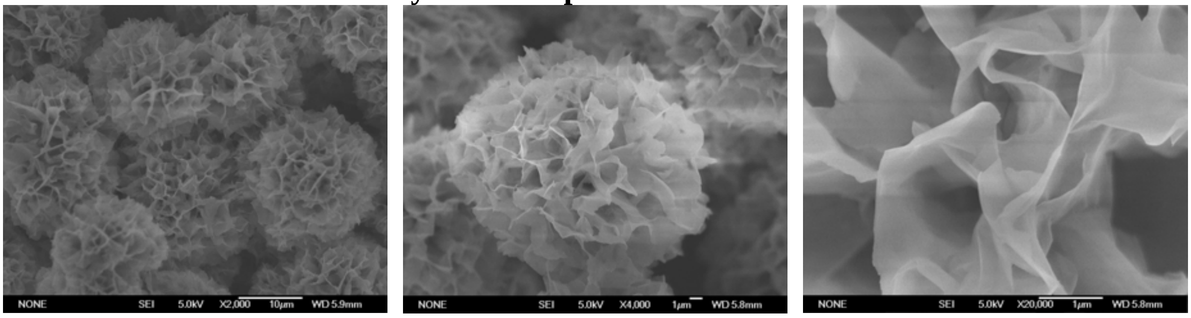

Argnine-incorporated nanoflower

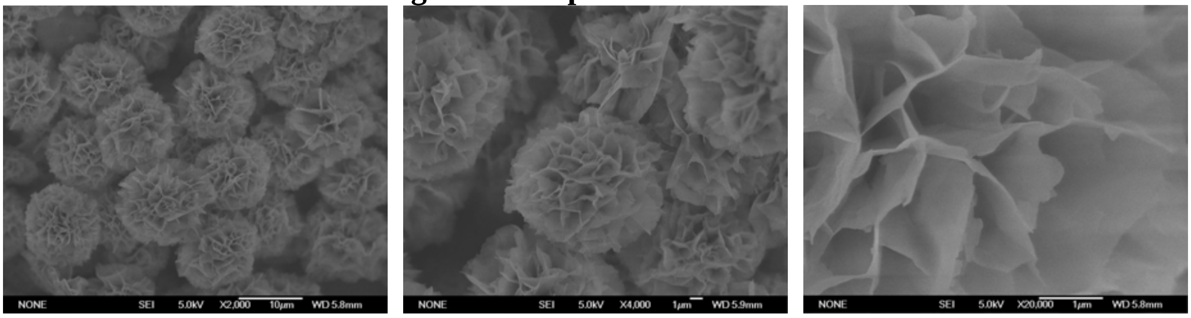

Aspartic acid-incorporated nanoflower

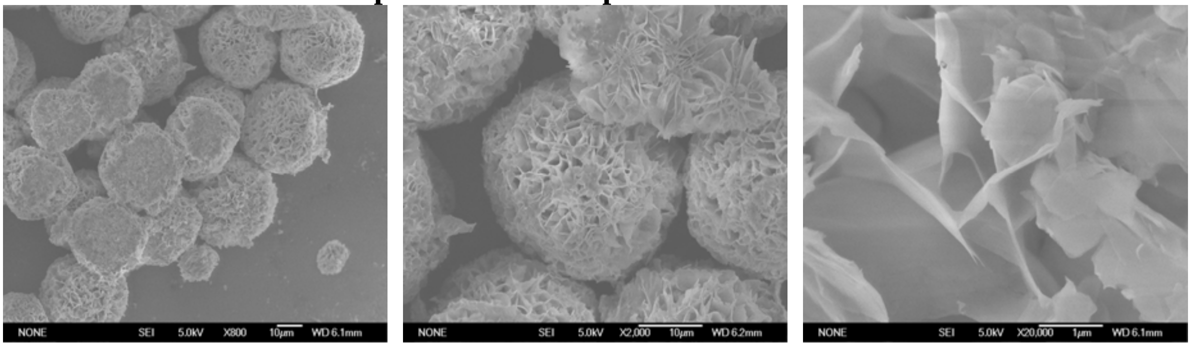

Glutamine-incorporated nanoflower

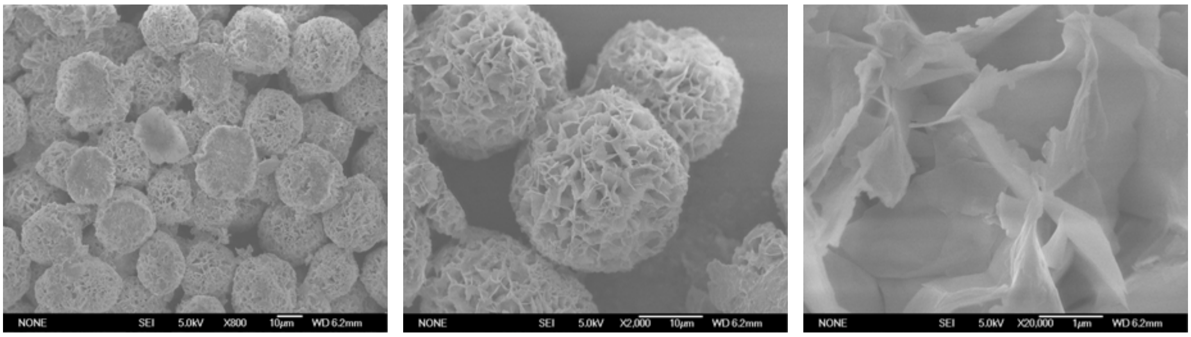

Glutamic acid-incorporated nanoflower

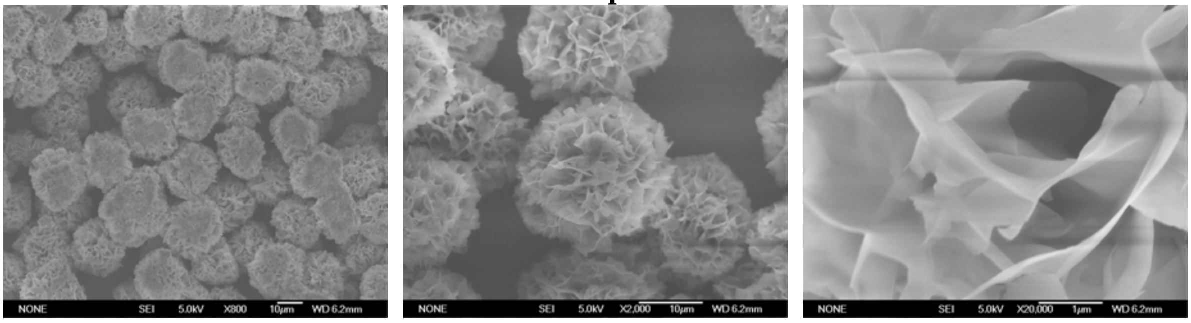

Leucine-incorporated nanoflower

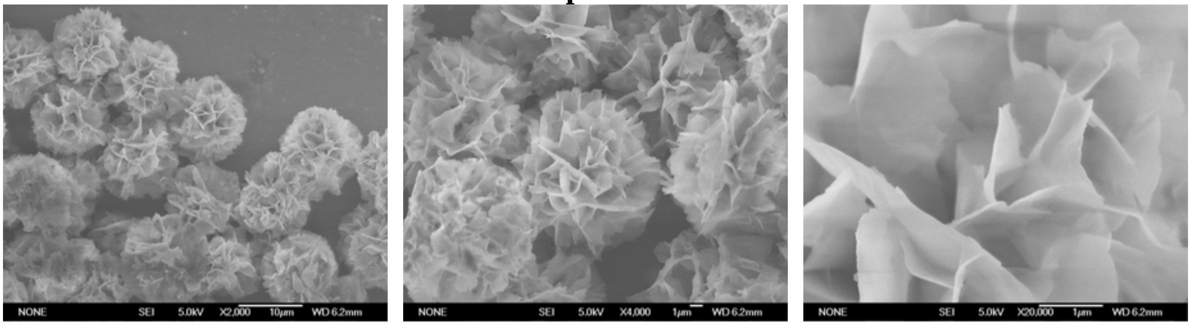

Methionine-incorporated nanoflower

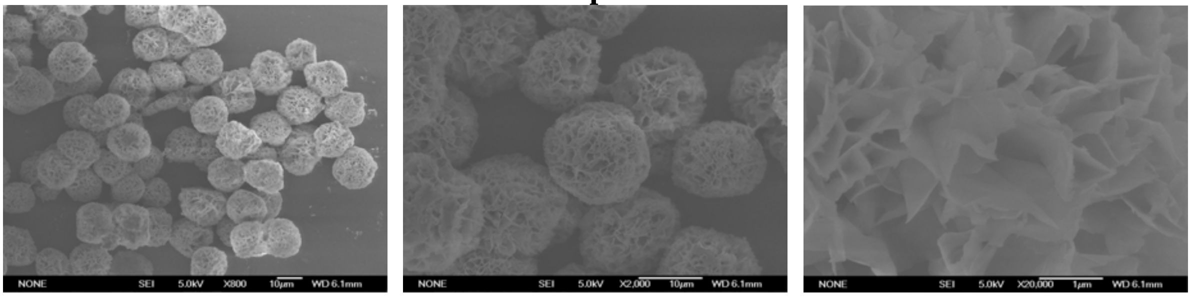

Serine-incorporated nanoflower

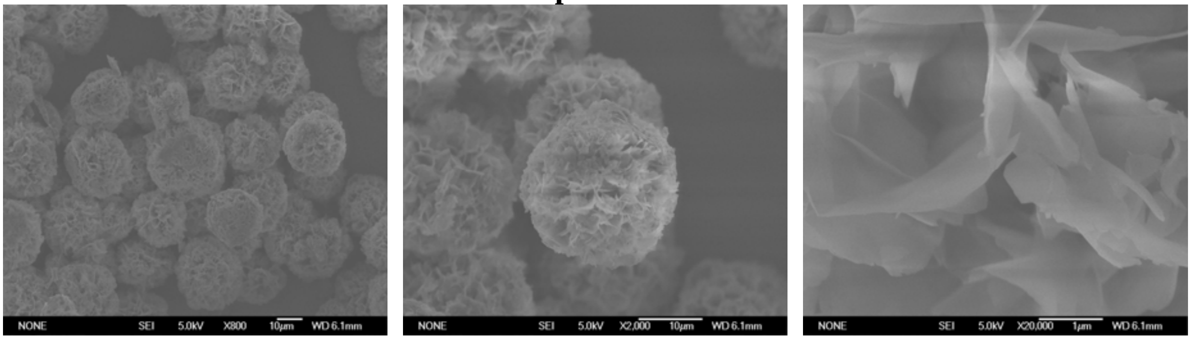

Phenylalanine-incorporated nanflower

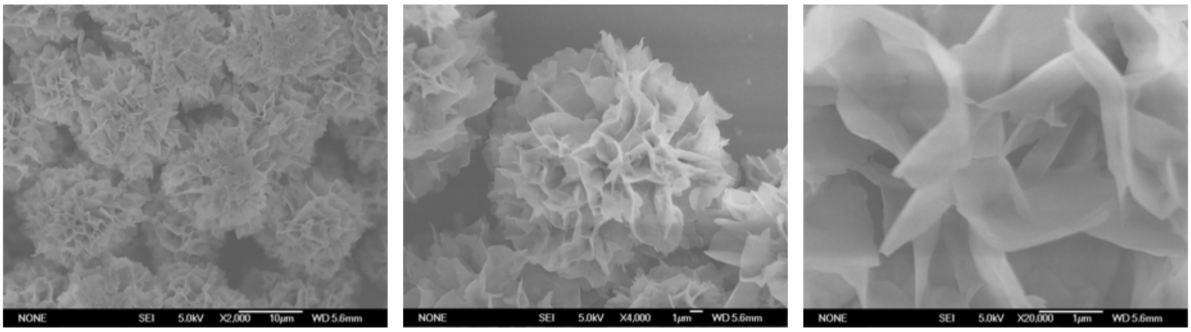

Isoleucine-incorporated nanoflower

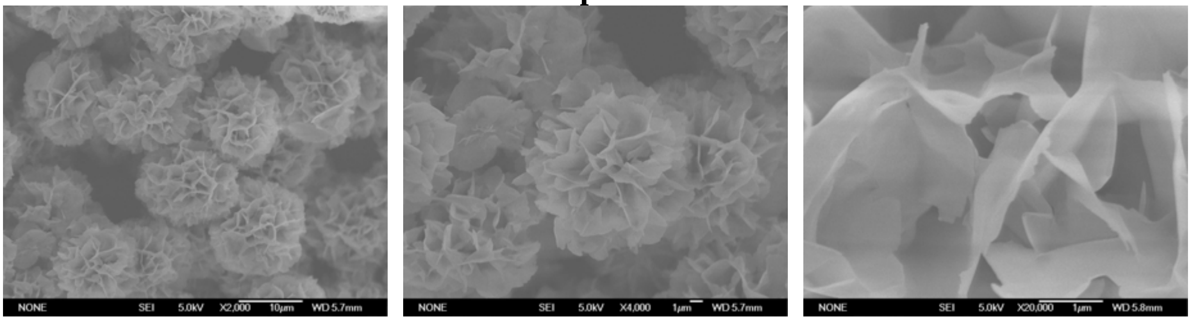

Tryptophane-incorporated nanoflower

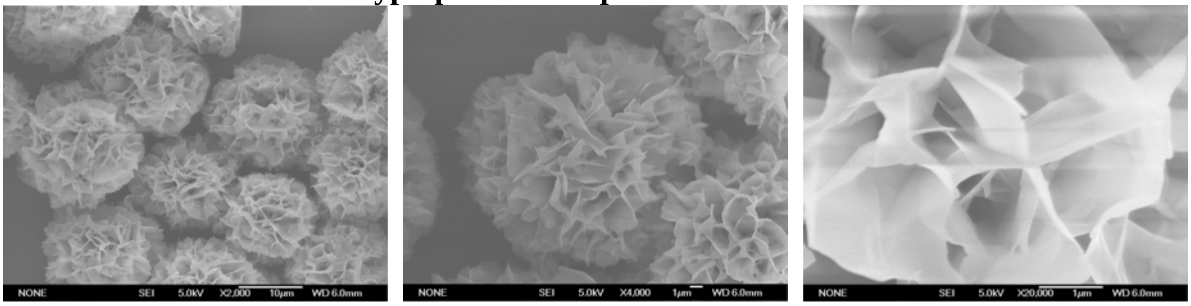

Threonine-incorporated nanoflower

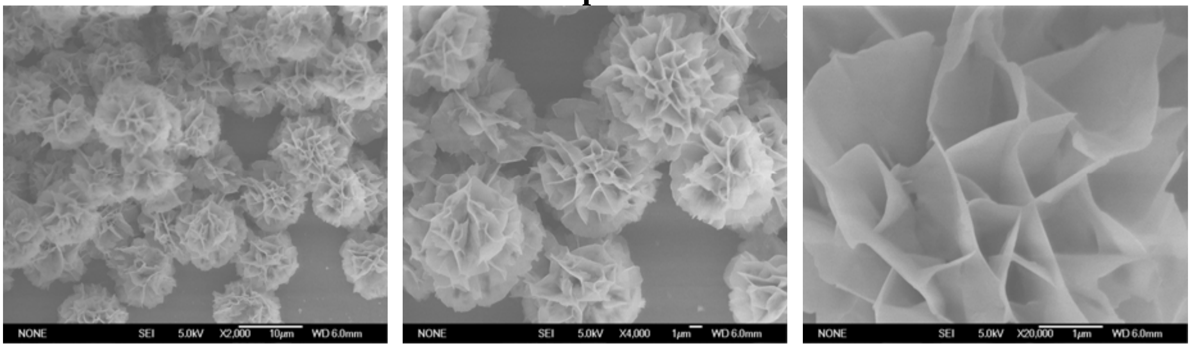

Alanine-incorporated nanoflower

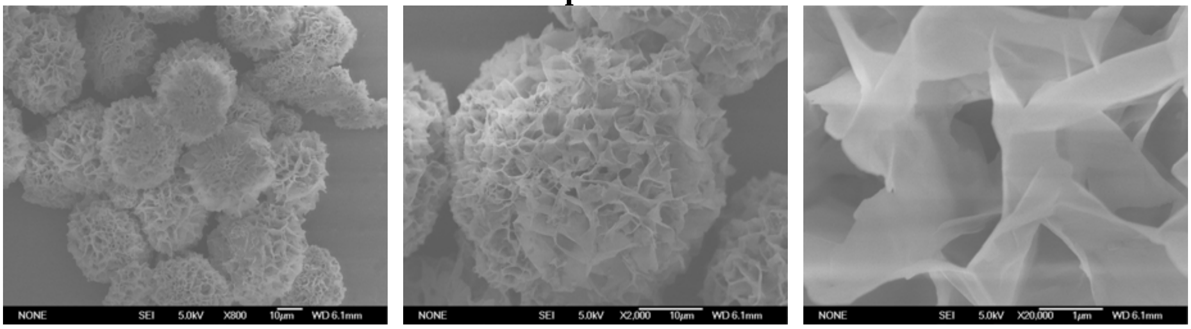

Lysine-incorporated nanoflower

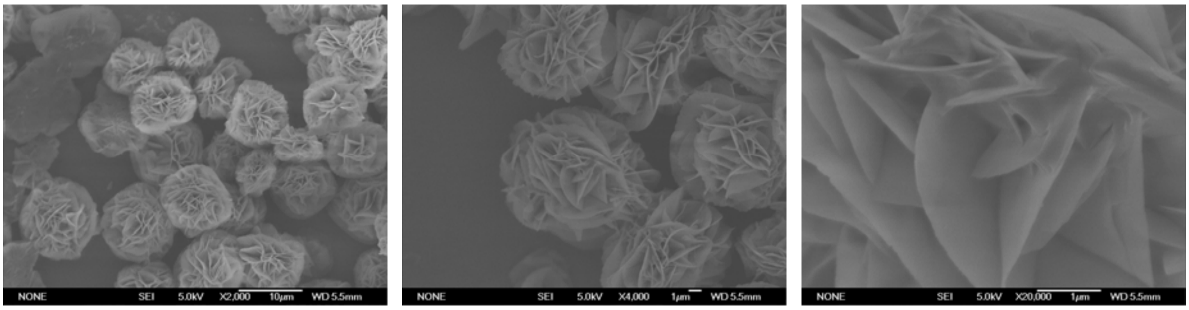

Histidine-incorporated nanoflower

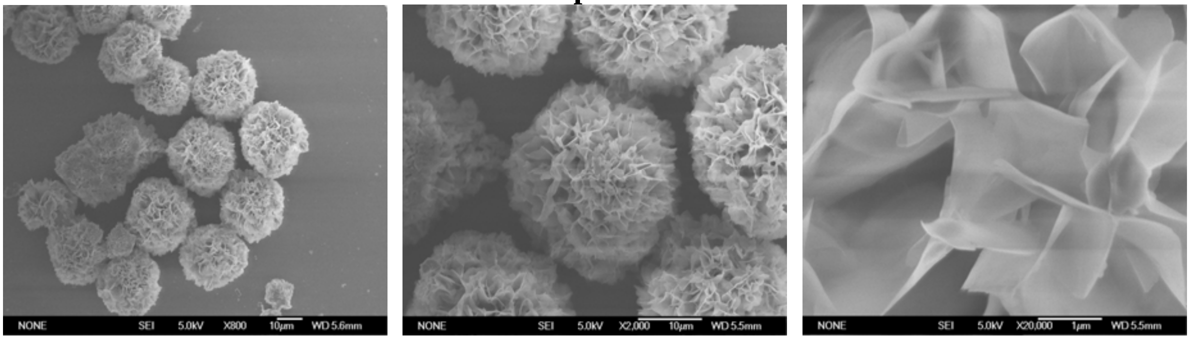

Tyrosine-incorporated nanoflower

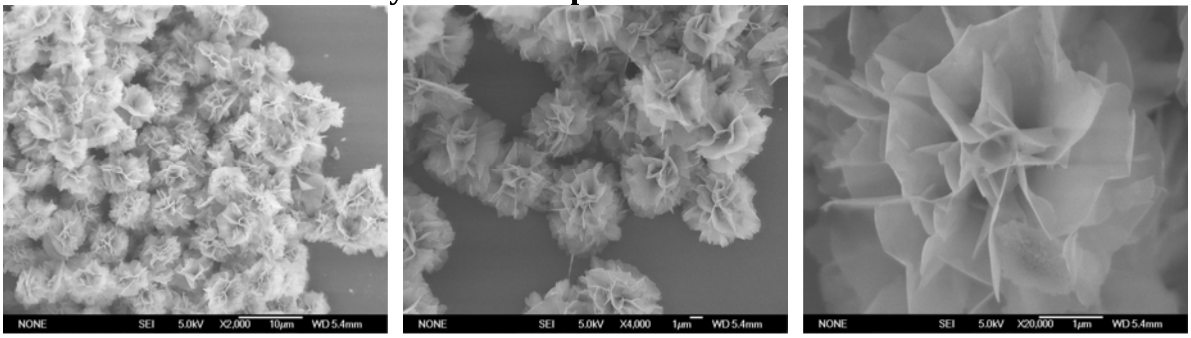

Valine-incorporated nanoflower

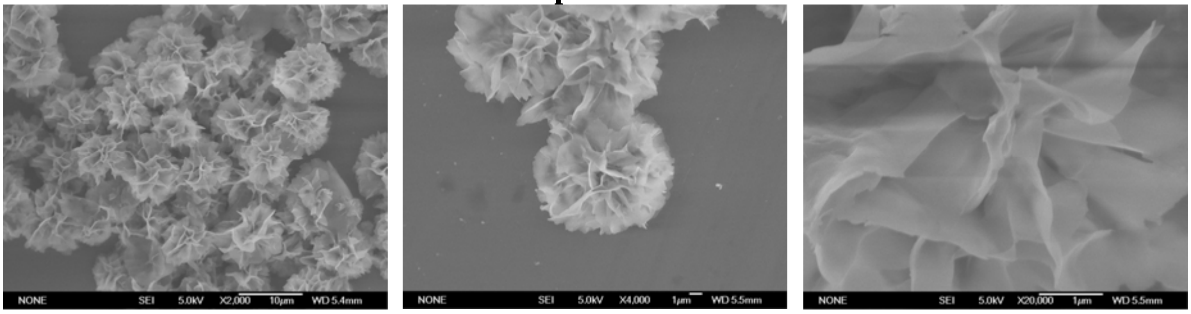

Proline-incorporated nanoflower

Figure S1. SEM of the hybrid organic–inorganic nanoflowers prepared from 19 natural amino acids.

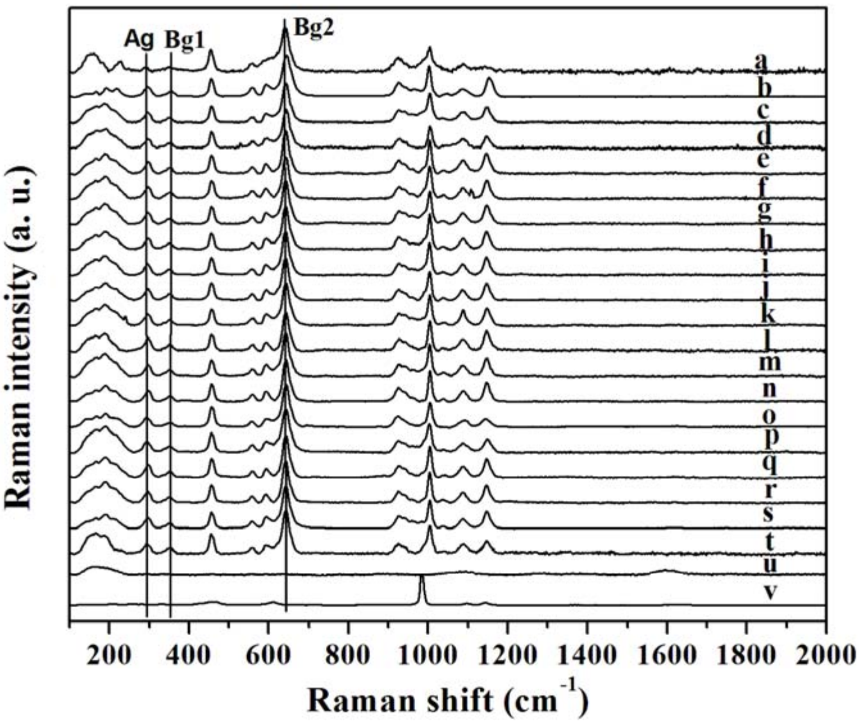

Figure S2. The Raman spectra of different amino acid-incorporated nanoflowers (a-t): (a) Asp, (b) Val, (c) Tyr, (d) Trp, (e) Thr, (f) Pro, (g) Phe, (h) Met, (i) Lys, (j) Leu, (k) Ile, (l) Gly, (m) Glu, (n) Gln, (o) Asn, (p) Arg, (q) Ala, (r) His, (s) Ser, (t) Cys. (u) $Cu_3(PO_4)_2$ and (v) $CuSO_4$.

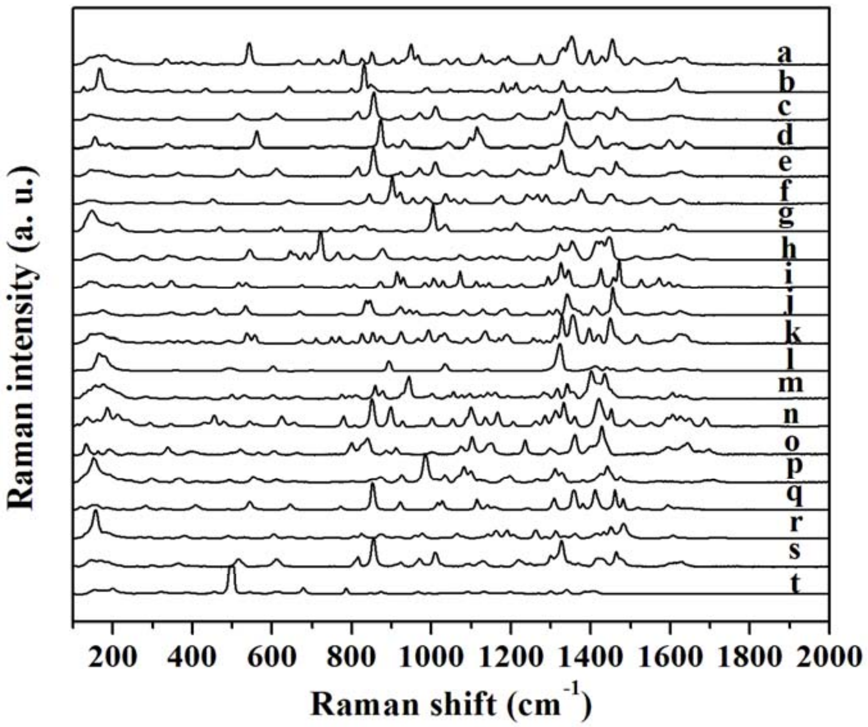

Figure S3. The Raman spectra of 20 natural amino acids (a-t): (a) Asp, (b) Val, (c) Tyr, (d) Trp, (e) Thr, (f) Pro, (g) Phe, (h) Met, (i) Lys, (j) Leu, (k) Ile, (l) Gly, (m) Glu, (n) Gln, (o) Asn, (p) Arg, (q) Ala, (r) His, (s) Ser, (t) Cys.

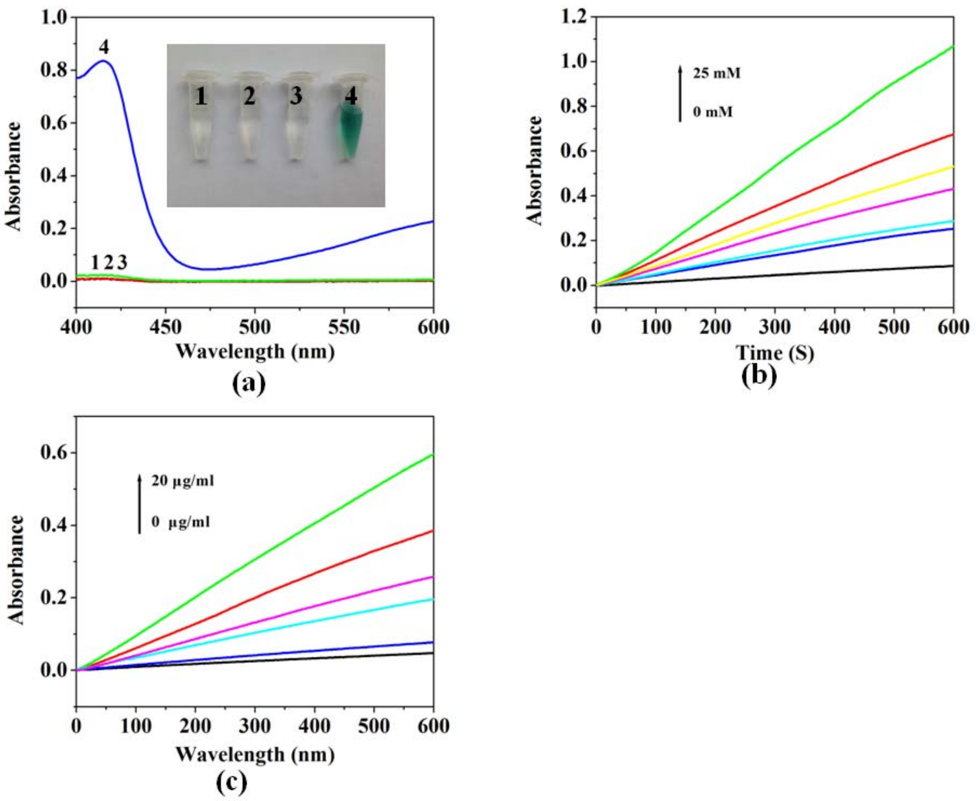

Figure S4. (a) absorbance curves of ABTS reaction solutions: (1) blank control, (2) only Asn-incorporated nanoflower, (3) only H_2O_2, (4) H_2O_2 and Asn-incorporated nanoflower; The inset is the photograph of corresponding solutions; (b) time-dependent absorbance changes of $ABTS^{\cdot+}$ with different concentrations of H_2O_2; (c) time-dependent absorbance changes of $ABTS^{\cdot+}$ with different concentrations of Asn-incorporated nanoflowers.

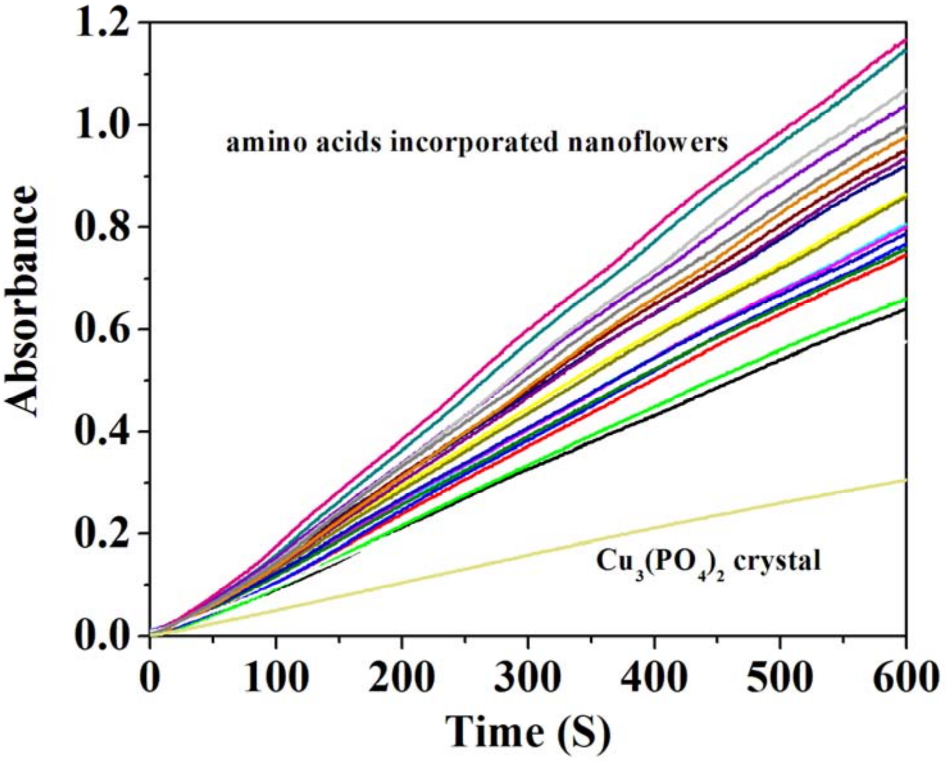

Figure S5. Time-dependent absorbance changes of ABTS$^{\cdot+}$ with different amino acid-incorporated nanoflowers or $Cu_3(PO_4)_2$ crystal in the presence of 25 mM H_2O_2.

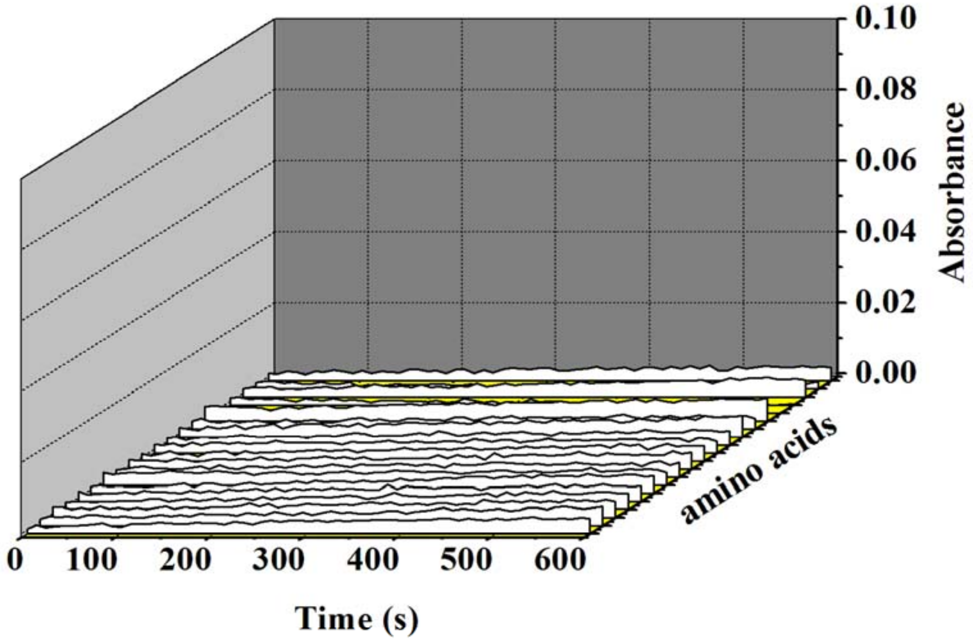

Figure S6. Time-dependent absorbance changes of $ABTS^{\cdot+}$ with different amino acids using Cu^{2+} ion as a catalyst in the presence of 25 mM H_2O_2.

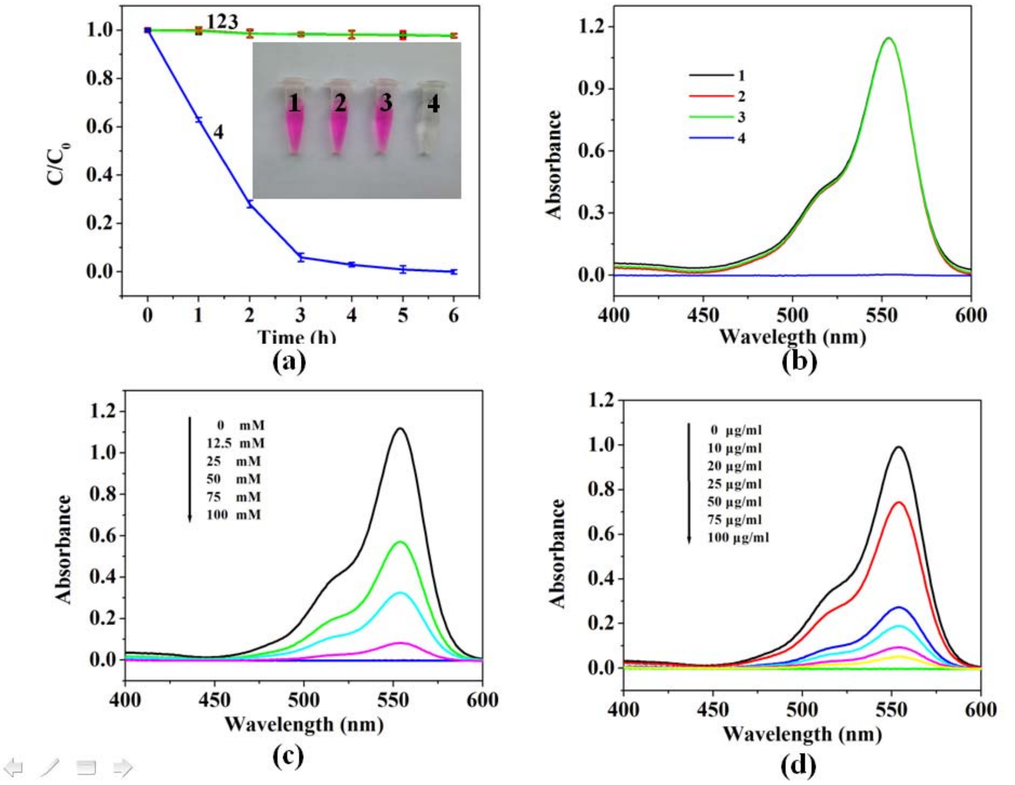

Figure S7. Removal of toxic organic compounds. (a) Rhodamine B degradation over time with different solutions: 1) blank control; 2) 20 μg/mL ASN-incorporated nanoflower only; 3) 100 mM H_2O_2 alone; 4) 20 μg/mL ASN-incorporated nanoflower and 100 mM H_2O_2. Inset was the typical photograph of corresponding solutions; (b) The absorption spectra of the Rhodamine B solution after incubated with different components for 6 h: 1) blank control; 2) 20 μg/mL ASN-incorporated nanoflower only; 3) 100 mM H_2O_2 alone; 4) 20 μg/mL ASN-incorporated nanoflower and 100 mM H_2O_2; (c) Relative absorbance spectra of the solution containing Rhodamine B and 20 μg/mL ASN-incorporated nanoflower with different concentrations of H_2O_2; (d) Relative absorbance spectra of the solution containing Rhodamine B and 100 mM H_2O_2 with different concentrations of ASN-incorporated nanoflower. All samples were incubated with 12.5 μg/mL Rhodamine B in phosphate buffer (pH 4.0, 10 mM) at 37 °C.

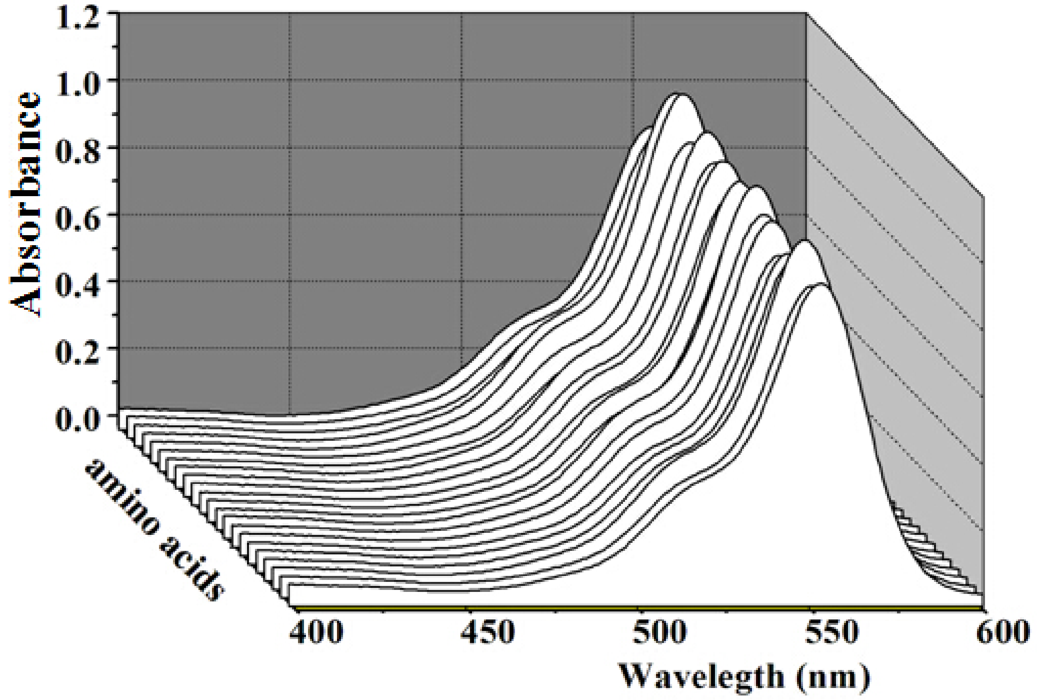

Figure S8. The absorption spectra of the Rhodamine B solution after incubated with different amino acids using Cu^{2+} ion as a catalyst for 6 h in the presence of 100 mM H_2O_2.

Table S1. Specific activity of different amino acid-incorporated nanoflowers and $Cu_3(PO_4)_2$ crystal using ABTS as substrate

Sample	Specific activity (μmol/mg/min)
Aspartic acid incorporated nanoflower	0.00799
Glutamic acid incorporated nanoflower	0.00801
Asparagines incorporated nanoflower	0.00889
Glutamine incorporated nanoflower	0.00897
Cysteine incorporated nanoflower	0.00901
Threonine incorporated nanoflower	0.01046
Serine incorporated nanoflower	0.01065
Glycine incorporated nanoflower	0.01073
Tyrosine incorporated nanoflower	0.01091
Phenylalanine incorporated nanoflower	0.01139
Tryptophan incorporated nanoflower	0.01145
Methionine incorporated nanoflower	0.01165
Proline incorporated nanoflower	0.01190
Isoleucine incorporated nanoflower	0.01231
Leucine incorporated nanoflower	0.01239
Valine incorporated nanoflower	0.01249
Alanine incorporated nanoflower	0.01332
Histidine incorporated nanoflower	0.01347
Arginine incorporated nanoflower	0.01367
Lysine incorporated nanoflower	0.01488
$Cu_3(PO_4)_2$ crystal	0.00457

Table S2. Specific activity of different amino acid-incorporated nanoflowers and $Cu_3(PO_4)_2$ crystal using Rhodamine B as substrate

Sample	Specific activity (μmol/mg/min)
Aspartic acid incorporated nanoflower	0.00717
Glutamic acid incorporated nanoflower	0.00721
Asparagines incorporated nanoflower	0.00800
Glutamine incorporated nanoflower	0.00805
Cysteine incorporated nanoflower	0.00809
Threonine incorporated nanoflower	0.00937
Serine incorporated nanoflower	0.00948
Glycine incorporated nanoflower	0.00963
Tyrosine incorporated nanoflower	0.00980
Phenylalanine incorporated nanoflower	0.01025
Tryptophan incorporated nanoflower	0.01030
Methionine incorporated nanoflower	0.01046
Proline incorporated nanoflower	0.01061
Isoleucine incorporated nanoflower	0.01172
Leucine incorporated nanoflower	0.01178
Valine incorporated nanoflower	0.01182
Alanine incorporated nanoflower	0.01189
Histidine incorporated nanoflower	0.01192
Arginine incorporated nanoflower	0.01200
Lysine incorporated nanoflower	0.01336
$Cu_3(PO_4)_2$ crystal	0.00406
